# Edward R. Roybal Centers for Translational Research in the Behavioral and Social Sciences of Aging

**DOI:** 10.1093/geroni/igaa057.1889

**Published:** 2020-12-16

**Authors:** Lisa Onken

**Affiliations:** National Institute on Aging, Bethesda, Maryland, United States

## Abstract

The goal of the Roybal Center program is the translation and integration of basic behavioral and social research findings into interventions to improve older people ‘s lives and the capacity of institutions to adapt to societal aging. Roybal Centers are structured within the conceptual framework of the multidirectional, translational NIH Stage Model to produce potent and implementable principle-driven behavioral interventions. The NIA’s Division of Behavioral and Social Research currently supports thirteen Roybal Centers, five of which have a primary focus on issues related to dementia care and caregiving support. Each Dementia Care Center has a unique focus that addresses issues such as social isolation, caregiving mastery, community-based resources and racial/ethnic minority health promotion. Additionally, a focus on the utilization of data science and behavioral economics related to palliative care, as well as a focus on the application of technology to improve assessments and interventions complete the scope of research endeavors.

